# Survey of Clinical and Pathological Characteristics and Outcomes of Patients With Prostate Cancer

**DOI:** 10.5539/gjhs.v6n7p49

**Published:** 2014-09-18

**Authors:** M. Alizadeh, S. Alizadeh

**Affiliations:** 1 Nephrology and kidney Transplant Research Center, Urmia University of Medical Sciences, Urmia, Iran; 2 Student medicines, Tehran University of Medical Sciences, Tehran, Iran

**Keywords:** pathological, characteristics, outcomes, prostate cancer

## Abstract

**Introduction:**

The importance of implementation: Prostate cancer is the most common malignancy in men and the second leading cause of cancer death in developed countries. Therefore, further studies about the protests of disease, diagnosis and timely treatment are essential.

**Study Method:**

In this study, 80 prostate cancer patients admitted to Imam Khomeini Hospital, Urmia in Iran from 2000 to 2008 were reviewed. Patients were studied according to their age, clinical protests, Gleason scoring, positive family history, smoking, type of treatment and post-treatment conditions. Questionnaires were adjusted based on the objectives and the data were extracted from the medical records of patients and the desired results were achieved.

**Results:**

In this study, the most common age group for prostate cancer is older than 60 years (92/5%). The most common type of pathology for prostate cancer is adenocarcinoma that 93.75% of cases are included. Secondary TCC with secondary source is present in 5% and sarcoma in 1.25% of cases. 46.25% of patients with prostate cancer are smokers. The most common clinical symptoms among patients are obstructive symptoms (56.25%), and irritation of the urinary tract (52.81%). Hematuria in 26.25% and urinary incontinence in 5% of cases have been recorded. 16.3% of patients referred with metastatic symptoms. Most patients with prostate cancer have Gleason score 5-7 (40%). All patients were undergoing prostatectomy (82.5% TURP and 17.5% SPP) and 47.5% of cases were bilateral orchiectomy. The cases reviewed, 22 were followed that included 27.5% of cases. Among them, 6 people have died due prostate cancer (27.27%) that the mean age of the patients after diagnosis until death was 34.4 months. 2 others died from other causes (9.09%). The remaining 14 cases were elder patients with a mean follow-up duration of 44 months.

**Conclusion:**

According to the results obtained in the present study, the most common type of prostate cancer pathology is adenocarcinoma that is included 93.75% of cases. Prostate sarcoma is present at 1.25% and secondary bladder TCC at 5% of cases more over the incidence of prostate sarcoma is reported in a lower age group than adenocarcinoma, which the results obtained in a similar study in Iran in other centers. Regarding the relationship between smoking and prostate cancer it can be considered one of the important factors in this regard.

## 1. Introduction

One of the male members, frequently involved by benign or malignant neoplasms is prostate gland. This gland included the greater part of the posterior urethral bulb. Anatomical location of the tumor is located in the true pelvis and at the anterior part separated from symphysis by the retzius space (Ramazani et al., 2011). Posterior surface of the prostate is separated from the rectal ampulla by denonvillier fascia. Base of the prostate continues by bladder neck and apex of the prostate is located on the upper surface of the orogenital diaphragm. From the side, prostate is connected with levator ani muscle (Ramazani et al., 2011). Prostate arterial blood supply comes from branches of the internal iliac artery. Vein drain takes place by the venous plexus. Innervation of this gland is by the pelvic neural network (Javadzadeh & Norouzian, 2000; Fathollahi, 1997; Nourouzi et al., 2004). McNeil in 1968, generalize the concept of regional anatomy for prostate. Gland volume is consists of 70% peripheral zone, 25% central zone and 5% interstitial zone in a young adult. 60-70% of prostate cancers originated from the peripheral zone, 10-20% from the interstitial area and 5-10% from the central region (Nourouzi et al., 2004; Nikoubakht, 1999; Fukagai, 2000; Fundacio, 1999; Gleason, 1992; Grignon, 1997).

### 1.1 Prostate Cancer

**Epidemiology and Incidence:** Prostate cancer is the most commonly diagnosed cancer and the second leading cause of dead (after lung cancer) in American men (Nikoubakht, 1999; Fukagai et al., 2000; Fundacio, 1999; Gleason, 1992; Grignon, 1997; Kurokawa et al., 2000; Mostofi & Sterhenn, 1992; Norrish et al., 1994; Patel, 2004; Ramos et al., 1999; Reuter, n.d.). Among all types of cancer, it is just prostate cancer that its incidence is increasing rapidly by age. The lifetime risk of hidden prostate cancer in a 50 year man (that is discovered incidentally on autopsy, not regarding the cause of death) is 40%; clinically evident prostate cancer is 9.5% and for deaths due to prostate cancer is 2.9%. Thus, many types of prostate cancer in a patient is trivial, while some types are more virulent and if they are diagnosed too late left untreated, will cause death. The broad spectrum of biological activity in prostate cancer makes it difficult for treatment of many patients (Nourouzi et al., 2004). Among the risk factors for prostate cancer is aging. Diagnosis of prostate cancer in men under 40 years is 1 out of each 10 thousand cases, for males 40-59 years is 1 out of each 103 cases and for males 60-79 years is 1 out of each 8 cases. There is higher risk for prostate cancer in African-Americans than whites. According to studies, a positive family history of prostate cancer increases its relative risk. Getting high-fat foods in the diet increases the risk by approximately 2%. Another factor is cadmium that is present in smoke alkaline batteries and welding. It is believed that previous vasectomy was a factor that increases the risk of prostate cancer, but this is still controversial (Javadzadeh & Norouzian, 2000; Nourouzi et al., 2004).

**Etiology:** Specific molecular mechanisms involved in the genesis of prostate cancer progression in terms of the laboratory are highly regarded. The gene responsible for familial prostate cancer is located on chromosome 1. Several regions of the human genome have been identified as hidden dungeons of tumor suppressor genes that may be involved in prostate cancer. The most common locations are 18q-8p-10q-13q-16q-17p chromosomes (Nourouzi et al., 2004).

**Pathology:** More than 95% of prostate cancers are adenocarcinomas (Reuter, n.d.). Of the remaining 5%, 90% of them are interstitial cell carcinoma and the remaining cancers are neuroendocrine carcinomas (small cell) or sarcoma. In the microscopic view, glands in carcinoma are not surrounded by collagen or scaffold cells and instead located “back to back” and apparently are completely separate from the primary scaffold. Neoplasm glands are carpeted by a layer of cubical cells with apparent nuclei. Layer of basal cells in the normal or hyper-plastic glands are not seen here. With increasing anaplasia, worn and irregular glandular structures, epithelium or pillar structures or screening or in severe cases pages of undifferentiated cells can be seen (Fathollahi, 1997; Mostofi, 1992). Neoplasia of epithelial prostate (PIN) is a precursor of invasive prostate cancer. Layer of basal cells of prostate gland buildings is not present in the cancer cases. When prostate cancer cytologic building is shaped by PIN, there is a layer of basal cells. PIN can be classified into two groups, high grade (HGPIN) and low grade (LGPIN). The clinical significance of this detection is that when the biopsy result of prostate with needle is HGPIN, usually close to 80% of cases may be associated with aggressive prostate cancer (Nourouzi et al., 2004).

**Grading and Staging:** Gleason grading system is the most commonly used system in the United States. In fact, this system is established based on the appearance of glandular structures under a microscope with low magnification. To determine the grade of the tumor, pathologists assign to a pattern of cancer that is seen more commonly primary grade and to the second most common pattern, secondary grade. The grades are from 1 to 5. If the full sample of cancer have one incidence pattern, both primary and secondary grades will be reported in one grade (Nourouzi et al., 2004). Summary of Gleason results are achieved from adding primary and secondary grades together. Gleason sum in well differentiated tumors is 2-4. Intermediate grade tumors have sum of 5-6 and in low- differentiated tumors is 8-10 ((Nourouzi et al., 2004). and 7). TNM staging system has been reported for prostate cancer. To the classification of the primary tumor (T) and clinical staging system using examination results with finger (DRE) and transrectal ultrasound (TRUS), but the biopsy results is not used. Another staging system is Witt Moore - Jute system (Nourouzi et al., 2004).

**Patterns of Progress:** Pattern of prostate cancer progression is fully defined. Likely to advance its position outside the prostate (extra capsular expand) increases with invasion to seminal vesicles and distance metastasis with increasing in tumor volume and reducing the degree of cancer differentiation (Nourouzi et al., 2004). Well-differentiated and small cancers (grades 1 and 2) are usually limited to prostate, whereas the massive cancers (4 cm^3^<) or with low grade differentiation (grades 4 and 5) in most cases have extended to the regional lymph node or bone or have metastasized. Cancer passes through the prostate capsule is a common phenomenon and often occurs through the space around the nerves. Invasion to the seminal vesicles shows the high probability of the existence of regional or distant disease. Prostate cancer that is locally advanced may be invasive to bladder trigone that may be causing urethral obstruction. Rectal conflict rarely occurs because the denonvillier fascia plays a role of a strong barrier. In most cases lymphatic metastases have been identified in lymph chain glands obturator (Nourouzi et al., 2004). The most common site of distant metastasis is the axial skeleton. Visceral metastases, lung, liver and adrenals are involved (Nourouzi et al., 2004; Reuter, n.d.).

**Clinical Findings:**

- Signs: most patients with primary stages of prostate cancer are without sign. Most of the disease symptom incidence indicates that the disease is locally advanced or has metastasized. Obstructive and irritative urinary symptoms may be occurring as a result of local tumor growth in the bladder trigone. Bone metastases can cause bone pain. Vertebral column metastatic by involving spinal cord may cause paresthesia and weakness in the lower limbs and incontinence of urine and stool (Nourouzi et al., 2004).
- Symptoms: DRE is essential in these patients. If the doctor found the prostate induration in examination, more assessment should be done (TRUS, PSA and prostate biopsy) (Nourouzi et al., 2004).
- Laboratory findings: maybe as the result of bilateral ureteral obstruction either due to direct progression of cancer to the bladder trigone or retroperitoneal adenopathy, azotemia may occur. Anemia maybe occurs in metastatic disease. In bone metastases alkaline phosphatase maybe increased.
- Indicators of cancer - prostate specific antigen - (PSA): Unfortunately, PSA is not specific for prostate cancer and increases in BpH and urethral manipulation. Sophisticated efforts have been done about PSA to detect prostate cancer that their main objective is to reduce the number of false positive tests. PSA velocity and PSA density are amongst other cases. Patients that their PSA levels increase greater than 0.75 ng/ml per year, it seems that the hidden cancer is more likely behind them. PSA density is PSA ratio to the tumor volume. PSA levels are increasing up to 0.12 ng/ml per gram of BPH tissue. Some researchers suggest prostate biopsy only when the density of PSA exceeded 0.1 or 0.15, and some believe that PSA density is not useful. PSA serum levels above 10 ng/ml are suggestive of a tumor with an origin of glands and ducts (Nourouzi et al., 2004; Sakr, 2000; Schwartz, 1999; Walsh & Retrik, 1998).

**Prostate Biopsy:** The sextet systematic biopsy prostate is the most common method used in detecting prostate cancer and mainly taken under TRUS guidance. Biopsy sites include top of the prostate, middle and base of the prostate on either side of the midsagittal halfway line between the lateral and medial prostate gland. Data obtained from sextet biopsies of prostate has focused primarily on the detection of cancer and is also used for staging (Nourouzi et al., 2004).

**Imaging:**

- TRUS: they are useful to get prostate biopsies and diagnosed cancer and to provide useful information for local staging. Almost all of the needle biopsies are derived from TRUS-guided prostate. If you perform this procedure, prostate cancer is more likely to show itself at the peripheral zone of prostate as hypoechoic lesions. Compared with DRE, TRUS method can be made more accurate local staging (Nourouzi et al., 2004).
- Endorectal MRI: reported staging accuracy is different from 51% to 92% by means.
- CT scan and MRI: These imaging are done to rule out metastasis to lymph nodes.
- Bone scan: presenting metastasis to surrounding, bone is usually involves. At the beginning of metastasis, soft tissues of metastasis are rare (Nourouzi et al., 2004).

**Molecular Staging:** Molecular staging is referred to determine prostate cell in the peripheral circulation of men who have prostate cancer (Nourouzi et al., 2004). In this method with the help of PCR (Polymerase Chain Reaction) the presence of RNA messenger is determined that is an indirect evidence for the presence of prostate cells in circulation. The clinical significance of positive results of this testing is currently unclear (Nourouzi et al., 2004).

**Screening for Prostate Cancer:** Most tumors that are identified by PSA can be treated and certain treatments are available. Many factors should be considered by doctors and their patients (patients who have been selected to screen for prostate cancer). Highest recommendation for screening is in certain populations that the disease incidence and death rates are higher, such as black men in America or men with a family history of prostate cancer. If the prostate cancer is diagnosed, the patient counseling will be conducted about the advantages and disadvantages of all available treatments, and follow-up care. The current data suggest that if the screening be done, would be better to perform DRE and PSA seroma at the same time (Nourouzi et al., 2004).

### 1.2 Treatment

**Localized Disease:**

1)- General remarks: suitable treatment for all stages of prostate cancer still remains as a big question. Typically, treatment decisions are founded on these bases: tumor grade and stage, life expectancy of the patient, the ability of any treatment to provide life without disease, related morbidity and patient preference and his doctor (Nourouzi et al., 2004).

2)- Follow-up and care: Patients who have prostate cancer are often older and may have associated diseases, as well as a prostate cancer that is usually small and well-differentiated and are found in the population have very slow growth rate. Case studies have shown that monitoring alone is an appropriate treatment for selected patients with prostate cancer. Even in this selected population, the death rate from cancer is nearly 10% (Nourouzi et al., 2004).

3)- Radical prostatectomy: the first prostatectomy of perineal was performed in 1904 by Hugh Hampton. However, this method due to its common side effects, such as incontinence and impotence remains uncommon. Revival of this method results in a better understanding of the surgical anatomy of the pelvis. The prognosis of patients treated with radical prostatectomy is associated with pathologic samples. A high percentage of patients with seminal vesicles involvement at radical prostatectomy, are infected distant disease. Further investigation have established nomograms to predict final pathological stage in radical prostatectomy that are based on PSA serum, DRE, clinical stage and Gleason sum based on biopsies (Nourouzi et al., 2004; Nikoubakht, 1999).

The role of neo-adjuvant hormonal therapy in men with localized prostate cancer is still under investigation. Patients with positive surgical margins in radical prostatectomy are still remaining controversial. All of these patients do not relapse moreover, selecting proper people to adjuvant radiation therapy remains as a problem (Nourouzi et al., 2004).

Morbidity that is associated with radical prostatectomy can be considerable and this morbidity is somehow related to the skill of surgeon. Immediate complications during surgery include, blood loss, damage to the rectum and urethra. Later complications include urinary incontinence and impotence (Nourouzi et al., 2004).

4)- Radiation therapy - treatment with external beam: external beam radiotherapy with conventional methods 6500-7000 cGy (XRT) radiation to the prostate (Nourouzi et al., 2004).

5)- Radiotherapy - Brachytherapy: renewal of interest to brachytherapy has been created due to technological advances that caused the possibility of replacing TRUS-guided radioactive seed.

Preliminary data in men with low volume and low-grade prostate cancer is promising, but randomized trials comparing brachytherapy with other forms of radiation therapy, and further studies are needed to establish the morbidity (Nourouzi et al., 2004).

6)- Surgical cryotherapy: in the past several years there recreated a willing to use seroma surgical in treatment of localized prostate cancer. Studies now show that at least in the short term, cryotherapy can cause negative surgical biopsy of the prostate after treatment and low PSA serum or undetectable. However, the morbidity associated with cryotherapy is significant and long-term results are unknown (Nourouzi et al., 2004).

**Locally Advanced Disease:** Radiation Therapy: Currently, most patients with prostate cancer, stage T3 with XRT following neo-adjuvant hormone therapy can be treated. The number of random testing has proved that this therapy is preferred to XRT method alone (Nourouzi et al., 2004).

**Recurrent Disease:**

1)- After radical prostatectomy: likelihood of cancer recurrence after radical prostatectomy is connected with cancer grade, pathological stage and the intracapsular progression. Cancer recurrence in patients with positive surgical margins, fixed intracapsular progression, invasion of seminal vesicles and disease in high-grade is more common (Nourouzi et al., 2004).

2)- After Radiotherapy: High levels of serum PSA after definitive radiotherapy represent a recurrence of their cancer. Prostate biopsy can determine local relapses. While bone scans and CT scans can identify distant relapses (Nourouzi et al., 2004).

**Metastatic Disease:**

1)- Primary endocrine therapy: since prostate cancer deaths almost invariably result in metastatic disease failure control, a big part of the research has focused on efforts to improve remote disease control. Most of the prostate carcinomas are hormone-dependent and close to 70-80% of men who have metastatic prostate cancer respond to different forms of androgen deprivation (Nourouzi et al., 2004).

2)- Early actions for failure on endocrine therapy: Patients receiving complete androgen block treatment that the increase in serum PSA levels shown are usually run by the anti-androgen withdrawal. Patients receiving monotherapy (only agonist LHRH or orchiectomy) that their serum PSA level began to raise that may be responding to adding an anti-androgen. Response rate in this situation is 20-30% (Nourouzi et al., 2004).

3)- Hormone refractory disease (Nourouzi et al., 2004). Given that the prostate cancer has the highest incidence (among men) and has high mortality rate and metastatic complications, more studies are essential to plan for proper screening, early diagnosis, early treatment depending on the patient’s condition and disease stage and secondary complication prevention.

## 2. Study Methods

This study is a retrospective - descriptive - cross sectional during the years 2000 to 2008 in Imam Khomeini Hospital, Urmia, Iran. All prostate cancer patients referred to Imam Khomeini Hospital, based on the records available, were studied. Detailed questionnaire based on the objectives of the study include: characteristics, clinical features, pathology type, treatment procedures performed, stage and grading of disease and status (follow-up) were adjusted. Data recorded cases of prostate cancer patients have been extracted that was admission to Imam Khomeini Hospital, Urmia. Receiving patients’ information and access, via telephone calls to track them in terms of survival and probably died during the period. After completing the questionnaires and data collection, statistical analysis of the data was attempted. Frequency of age with prostate cancer, clinical symptoms, smoking, family history, DRE results, pathology type, Gleason scoring, remedial action taken and current status of patients were determined and drawn graphs and tables and results were obtained. SPSS 15 software is used for data analysis (Kaveh et al., 2013).

## 3. Results

80 completed questionnaires related to patients with prostate cancer in Imam Khomeini Hospital from 2000 to 2008 in terms of age group less than 40 years, 40 to 60 years and above 60 years were divided and frequency of prostate cancer was investigated. Frequency of patients in the age group less than 40 years was 2 cases equal 2.5%. In the age group 40 to 60 years, the frequency was 4 cases equal 5%. Aged over 60 years, the highest frequency was seen in patients with prostate cancer that was 74 cases equal 92.5% (Table 1). The mean age of patients was 70.3 years.

**Table 1:** Frequency of prostate cancer according to age groups

Age groups	Frequency	Percentage
Less than 40 years	2	5.2%
40-60 years	4	5%
More than 60 years	74	5.92%

The most common age group was more than 60 years equal 92.5%. Among the 80 patients with prostate cancer 37 cases, 46.25% of patients were smokers and 43, 53.75% were non-smokers. Among the 37 smokers with cancer, 21 patients, 56.75% were heavy smoker (over 20 p/y) and 16 patients, 43.25% were non-heavy smoker. Symptoms of prostate cancer were completed separately in the questionnaire and were divided as different categories in terms of irritative symptoms and urinary obstruction, urinary incontinence, hematuria and metastatic symptoms. According to the results urinary irritative symptoms were present (dysuria, urinary iteration, nocturia, urinary urgency) in 42 cases out of 80 cases with 52.81% of patients. Urinary obstruction (dribbling, double voiding, effort to urination, urinary retention, feeling inadequate bladder drain, decreasing diameter and pressure of urinate and pauses during urine) were recorded in 45 cases out of 80 cases with 56.25%. Urinary incontinence was reported in 4 cases examined (5%). Hematuria was recorded in 21 out of 80 cases with 26.25%. Metastatic symptoms (bone pain, weight loss, abnormal bowel movements, symptoms of kidney failure, paresthesia and weakness of the extremities, lymphedema of the lower extremities, and lymphadenopathy regional) were recorded in 13 out of 80 cases with 16.3%. (Table 2)

**Table 2:** Frequency of clinical symptoms in patients with prostate cancer

Clinical symptoms	Frequency	Percentage
Irritative urinary symptoms	42	81.52%
Urinary obstruction symptoms	45	25.56%
Urinary incontinence	4	5%
Hematuria	21	25.26%
Metastatic symptoms	13	3.16%

According to the results obtained in patients with prostate cancer, urinary incontinence, irritative urinary symptoms and urinary obstruction symptoms are the most common. According to the results of the pathology reports in 75 cases out of 80 prostate cancers was of adenocarcinoma kind with 93.75%. 4 cases with secondary interstitial cell carcinoma (bladder origin) equal to 5% were reported (Table 3). One case of sarcoma (rhabdomyosarcoma) was recorded that include 1.25%. Frequency of neuroendocrine carcinoma and LGPIN-HGPIN intraepithelial carcinoma were zero.

**Table 3:** Different prostate cancers based on the type of pathology

Type of pathology	Frequency	Percentage
Adenocarcinoma	75	75.93%
Interstitial cell carcinoma	4	5%
Neuroendocrine carcinoma	0	0
Sarcoma	1	25.1%
HGPIN	0	0
LGPIN	0	0

The most common type of prostate cancer is adenocarcinoma with 93.75% of cases. Of 80 cases investigated in 40 cases (50%) Gleason grading was not recorded. In 40 cases remaining that had Gleason grading 14 cases had 2-4 Gleason score equal to 35% (tumor with high differentiation grade). 16 cases equal to 40% had 5-7 Gleason score (moderately differentiated). 10 cases equal to 25% had 7-10 Gleason score (low grade differentiation) (Table 4).

**Table 4:** Gleason scoring among patients surveyed

Scoring	Frequency	Percentage
Unrecorded cases	40	50%
Score 2-4	14	35%
Score 5-7	16	40%
Score 7-10	10	25%

The most common Gleason scoring is in patients with 5-7 score, so prostate cancer is in its moderate differentiation. Of 80 patients with prostate cancer in 74 cases, 92.5% DRE results were recorded. Of the 74 patients recorded DRE 10 cases had a normal prostate examination, 13.5%. The frequency of prostate more than +1 was 9 cases, 12.16%. DRE result at +2 in 30 cases was equal to 40.54% and DRE result at +3 in 24 cases was equal to 32.43%. DRE result at +4 was equal to 1.35%. In 10 cases, 13.5% in rectal exam for nodularity prostate and induration was observed. In the cases studied, the induration, nodularity and abnormality of prostate that is to be expected in these patients has not been mentioned and documented examination findings are based on an internship or residency that is expected to be more realistic and accurate. Closer examination and registration of DRE is essential. Although DRE examination results are largely subjective and interpersonal changes by the same individual is also significant. All patients underwent prostatectomy. Serum PSA levels were noted in 24 cases (30%) (Table 5).

**Table 5:** DRE results of patients surveyed

DRE result	Frequency	Percentage
Normal	10	5.13%
1+	9	16.12%
2+	30	54.40%
3+	24	43.32%
4+	1	35.1%
Nodularity and induration	10	5.13%

DRE 2 + result were in 40.54% of cases. And induration in 13.5% of cases has been recorded. Of 80 cases reviewed, there was one positive family history of prostate cancer, 1.25%. There was a positive family history in the father and the brother. Of 80 cases studied all was underwent prostatectomy (100%). 66 cases out of 80 cases, 82.5% underwent transurethral prostatectomy (TURP) and 14 out of 80 cases, 17.5% underwent open prostatectomy surgery (SPP). In addition, 38 cases underwent bilateral orchiectomy surgery, 47.5%. Cases of chemotherapy, radiation therapy and other therapeutic interventions were not recorded. Of 80 cases reviewed, only 22 cases were followed up with respect to the information contained in the file, 27.5%. Of followed up cases 21 patients, 95.45% underwent TURP and 1, 4.5% underwent SPP. 15 patients, 68.18% underwent bilateral orchiectomy. Among the 22 patients followed, 6 deaths from prostate cancer, 27.27% were obtained. The average life expectancy after diagnosis was 34.4 months. 2 patients had died due to other causes. 14 out of 22 cases were healthy (63.6%) that their follow-up was 44 months (Table 6).

**Table 6:** Percentage of patients followed up

	Frequency	Percentage
Followed up patients	22	5.27%
Healthy	14	63.63
Healthy patients who have bilateral orchiectomy	11	57.78%
Death from prostate cancer	6	27.27%
Death cases who had orchiectomy	4	66.66%
Death because of other causes	2	09.9%

27.27% of the cases treated have died of prostate cancer. Average life expectancy of patients after diagnosis until death was 34.4 months.

## 4. Discussion

Prostate cancer is the most common malignancy in men and the second leading cause of cancer death in developed countries. Among the malignancies it is the prostate cancer that its incidence increases with age very quickly. According to studies, a positive family history and smoking increase the risk of prostate cancer. Most patients who are diagnosed with prostate cancer have no symptoms. The incidence of symptoms are often indicated local progression or metastatic. TUR prostate tumors detection methods are sextet needle biopsy, TRUS, FNA and cytology. Treatment, depending on the clinical and pathological stage, is different. In some cases due to high levels of illness and very high age of the patient and palliative care, and in other cases, depending on the condition diagnosed, radical prostatectomy, radiation therapy, chemotherapy, hormone therapy take place. In this study, the most common prostate cancer is patients older than 60 years that include 92.5% of cases. These findings have been mentioned in authoritative urology references, such as Smith Urology. In research conducted by Dr. Javadzadeh and Dr. Norouzian at Shahid Beheshti Medical Center, University of Medical Sciences, the highest rate of prostate cancer was patients older than 60 years (Javadzadeh & Norouzian, 2000). According to the results obtained in the present study, the most common type of prostate cancer pathology is associated to adenocarcinoma that includes 93.75% of cases. Prostate sarcoma is present in 1.25% and secondary bladder TCC in 5% of cases. In addition, prostate sarcoma has been reported in lower age group than adenocarcinoma that the results in similar studies in other centers in Iran are also obtained (Javadzadeh & Norouzian, 2000; Fathollahi, 2004; Nourouzi et al., 2004).

Regarding the relationship between smoking and the risk of prostate cancer 46.25% of patients are smokers that 56.75% of them are heavy smoker. For the study, the highest correlation between smoking and the incidence of prostate cancer was in the secondary TCC and then about 40% of patients with adenocarcinoma were smokers. According to the results obtained in the present study, the clinical symptoms of prostate cancer, the most common symptoms among patients were irritative symptoms (52.81%) and urinary tract obstruction (56.25%); urinary incontinence was recorded in 5% and hematuria in 26.25%. About 16.3% of patients were referred with metastatic symptoms. These findings are somewhat similar to results of another study conducted in Iran (Fathollahi, 2004; Nourouzi et al., 2004).

In a recent study about DRE results in 32.43% of the cases reviewed prostate is bigger than normal, and is + 3, and in 40.54% of the cases prostate is + 2. In 13.5% of cases prostate induration is also reported. The Gleason scoring for prostate cancer patients in the current study, the most frequent Gleason score is 5-7. Due to this problem, most cases are at the high level of disease. In research conducted by Dr. Javadzadeh and Dr. Norouzian pointed the late reference of patients to upper stages of the disease during diagnosis (Javadzadeh & Norouzian, 2000). In a study conducted by Ramos cg, Smith ds et al. also noted that higher Gleason score and recurrence rate is higher in the upper stages of the disease. And upper stages of the disease are also seen in upper ages. In a recent study, all patients with prostate cancer have undergone prostatectomy. (100%) and 38 cases, equal to 47.5% underwent bilateral orchiectomy. All patients presented with symptoms were referred and underwent prostatectomy. While the recommendation is that all patients initially should be measured accurate PSA and DRE and, where necessary, sextet biopsy done and after confirming prostate cancer, according to the stage, perform appropriate and common therapies in advanced centers, such as radical prostatectomy. Unfortunately because of the lack of sextet biopsy facilities in this province and patients unwilling to bear the costs of travel to other centers, patients with prostate cancer is still in this center are treated with the usual methods of treatment for many decades, and radical prostatectomy is still an unknown treatment. The results obtained in patients treated for prostate cancer, 22 out of 80 cases were followed up from that include 27.5% of cases. Among these cases there are 8 death cases that include 36.3% of cases, among the death cases, 6 cases of death were from prostate cancer (27.27%). Average life expectancy for patients after diagnosis is 34.4 months. The other 2 patients died of myocardial infarction and cerebral hemorrhage. As in a similar study conducted in Iran, in our country, patients are diagnosed and treated at the upper stages of the disease (Javadzadeh & Norouzian, 2000, Fathollahi, 1997, Nourouzi et al., 2004). In Ramos cg; Smith ds et al. study also high recurrence rates in upper stages of disease and death caused by it have been reported (Ramos et al., 1999).

## 5. Recommendations

According to the results of the present study, proper program for screening individuals at high risk prostate cancer (PSA level measurement and detailed examinations including DRE) and enhance diagnostic methods with respect to existing facilities in developing countries (including sextet biopsy under TURS) and proper care after diagnosis and regular follow-up of patients to reduce morbidity and mortality and cancer complications recommended.
